# Reassessing Benign *ASXL1* Variants in Bohring–Opitz Syndrome: The Role of Population Databases in Variant Reinterpretation

**DOI:** 10.3390/genes17020231

**Published:** 2026-02-12

**Authors:** Liliana Fernández-Hernández, Sergio Enríquez-Flores, Nancy L. Hernández-Martínez, Melania Abreu-González, Esther Lieberman-Hernández, Gerardo Rodríguez-González, Sinuhé Reyes-Ruvalcaba, Miriam E. Reyna-Fabián

**Affiliations:** 1 Laboratorio de Biología Molecular, Instituto Nacional de Pediatría, Secretaría de Salud, Mexico City C.P. 04530, Mexico; dralilianafernandez@gmail.com (L.F.-H.); biol.nancyhdz@gmail.com (N.L.H.-M.); 2 Laboratorio de Biomoléculas y Salud Infantil, Instituto Nacional de Pediatría, Secretaría de Salud, Mexico City C.P. 04530, Mexico; sergioenriquez@ciencias.unam.mx; 3 Laboratorio Genos Médica, Mexico City C.P.06700, Mexico; dra.melaniaabreu@gmail.com; 4 Centro Médico ABC, Mexico City C.P. 05300, Mexico; 5 Departamento de Genética Humana, Instituto Nacional de Pediatría, Secretaría de Salud, Mexico City C.P. 04530, Mexico; estherlieberman@yahoo.com.mx; 6 Subdirección de Servicios Clínicos, Genética, Hospital Regional de Alta Especialidad de Zumpango, Zumpango C.P. 55600, Mexico; gerarodgo@gmail.com; 7 Universidad Cuauhtémoc, Campus Querétaro, Querétaro C.P. 76060, Mexico; sinuhe.reyruv@gmail.com

**Keywords:** *ASXL1*, Bohring–Opitz syndrome, VUS classification, protein modeling, clonal hematopoiesis of indeterminate potential (CHIP)

## Abstract

**Background/Objectives**: *ASXL1* is a chromatin-associated gene implicated in both hematologic malignancies and neurodevelopmental disorders, including Bohring–Opitz syndrome (BOS). Although many *ASXL1* variants are well classified, a substantial proportion remain variants of uncertain significance (VUS), complicating molecular diagnosis and genetic counseling. The objective of this study was to evaluate whether structural context can inform the interpretation of selected *ASXL1* missense variants in a clinical setting. **Methods**: We describe a 17-year-old female with clinical features consistent with BOS carrying the heterozygous *ASXL1* variant p.Q1448R, currently classified as benign under ACMG/AMP guidelines. Three-dimensional in silico structural modeling was performed using AlphaFold3 and available crystallographic data. Three additional *ASXL1* missense variants classified as VUS in ClinVar (p.R265H, p.T297M, and p.Y358C) were also analyzed. Evolutionary conservation, domain localization, and residue-level interactions were assessed. **Results**: Structural modeling indicated that the p.Q1448R substitution alters polar interactions and introduces a steric constraint near a conserved PHD-type zinc finger domain. Variants p.R265H and p.T297M affected stabilizing interactions within the DEUBAD, which is involved in BAP1 activation, while p.Y358C altered a polar microenvironment adjacent to a chromatin-interacting region. All analyzed variants, except p.T297M, localized to evolutionarily conserved regions. **Conclusions**: This study demonstrates that in silico structural analysis can provide complementary, domain-level insights for the interpretation of *ASXL1* missense variants that remain classified as benign, likely benign or VUS under current frameworks. Such approaches may assist in prioritizing variants for further functional evaluation and refining molecular interpretation when experimental data are limited.

## 1. Introduction

Next-generation sequencing (NGS) technologies have greatly expanded the identification of genetic variants underlying monogenic disorders; however, the interpretation of missense variants remains a major challenge in clinical genomics. The publication of the American College of Medical Genetics and Genomics and Association for Molecular Pathology (ACMG/AMP) guidelines in 2015 provided a standardized framework for variant classification [1]. Despite this advance, some criteria remain ambiguous, leading to variability in interpretation and low concordance among diagnostic laboratories [2]. Subsequent efforts, including the Sherloc framework, quantitative Bayesian models, and disease-specific guidelines, have refined variant interpretation and improved reproducibility [3,4,5,6,7]. Nevertheless, a substantial proportion of variants—particularly missense changes—remain classified as variants of uncertain significance (VUS), underscoring persistent gaps between computational prediction, population-based evidence, and biological effect.

Beyond interpretative frameworks, population databases have become essential tools in variant classification. The GnomAD database, for example, compiles variant frequencies from over 730,000 exomes and 76,000 genomes (v4.1 dataset; GRCh38, [8]), derived from healthy, unrelated individuals of diverse ancestries [9]. However, it is crucial to recognize that not all variants in public databases are of germline origin; some may be somatic, particularly in genes frequently associated with clonal hematopoiesis of indeterminate potential (CHIP), such as *ASXL1*, *TET2*, *DNMT3A*, *PPM1D*, *JAK2*, *SF3B1*, *SRSF2*, *TP53*, *SETBP1*, *PTPN11*, and *NRAS* [10,11,12]. This distinction is especially relevant for interpreting variants in individuals over 40 years of age, where the presence of somatic mutations may mimic germline pathogenic variants responsible for autosomal dominant or autosomal recessive conditions [13,14]. For genes with dual roles in developmental disorders and age-related clonal expansion, such as *ASXL1*, reliance on population frequency alone may therefore lead to variant misclassification.

The *ASXL1* gene (Additional Sex Combs Like 1; MIM*612990, 20q11) encodes a protein that is a core component of the polycomb repressive deubiquitinase (PR-DUB) complex. This complex includes BAP1, MBD5/6, and other accessory proteins and is involved in chromatin remodeling through histone H2A deubiquitination [15,16,17]. The ASXL1 protein contains conserved domains such as the DEUBAD (which binds BAP1 and is essential for complex assembly and activation), and a PHD-type zinc finger domain, implicated in recognizing post-translational histone modifications and mediating transcriptional regulation [18,19].

Somatic mutations in *ASXL1*, particularly frameshift or nonsense variants, are frequently observed in hematological malignancies, including myelodysplastic syndromes (MDS), myeloproliferative neoplasms (MPN), chronic myelomonocytic leukemia (CMML), and acute myeloid leukemia (AML), with haploinsufficiency likely being the main pathogenic mechanism [20]. In contrast, germline variants in *ASXL1* cause Bohring–Opitz syndrome (BOS; MIM#605039), an ultra-rare (1:1,000,000) [21,22] autosomal dominant neurodevelopmental disorder characterized by intrauterine growth restriction, intellectual disability, facial dysmorphism, hypertrichosis, and a distinctive BOS posture. Although BOS has classically been associated with truncating variants, accumulating evidence indicates that missense variants may contribute to atypical or milder phenotypes, expanding the recognized clinical and molecular spectrum of the disorder.

Although approximately 125 pathogenic or likely pathogenic *ASXL1* variants associated with BOS have been reported in ClinVar [23], a substantial proportion of reported variants remain classified as benign/likely benign or VUS, despite suggestive clinical features. Moreover, the presence of atypical phenotypes and under-recognition of BOS in clinical settings increases the risk of misdiagnosis, underscoring the importance of integrative approaches that extend beyond categorical variant classification.

Recent advances in protein structure prediction and large-scale functional genomics have highlighted the importance of structural context in missense variant interpretation. Studies generating comprehensive functional “landscapes” of coding variation have demonstrated that variants affecting conserved structural microenvironments or interaction interfaces may exert functional effects that are not captured by traditional in silico predictors or population-based filters. These findings support the use of three-dimensional structural modeling as a complementary, hypothesis-generating tool for variant interpretation, particularly in complex chromatin regulators such as ASXL1.

Here, we report a 17-year-old female with clinical features consistent with BOS carrying the heterozygous missense *ASXL1* variant NM_015338.6:c.4343A>G (p.Q1448R), currently classified as benign under ACMG/AMP criteria and Bayesian interpretation [1,4]. While these classification frameworks rely in part on population frequency data, it is increasingly recognized that such datasets may include somatic variants, particularly in genes associated with clonal hematopoiesis, which can complicate the interpretation of germline pathogenicity in certain contexts. To investigate the structural context and potential functional implications of this variant, we performed three-dimensional protein modeling of p.Q1448R together with three additional *ASXL1* missense variants classified in ClinVar as likely benign or VUS (p.R265H, p.T297M, and p.Y358C), focusing on their localization within conserved functional domains. Through this approach, we aim to illustrate how integrative structural analyses can complement existing variant interpretation frameworks and help contextualize uncertain missense variants in clinically relevant genes.

## 2. Materials and Methods

### 2.1. Case Presentation

We report a 17-year-old female patient presenting with short stature (HP:0004322), intellectual disability (HP:0010864), synophrys (HP:0000664), exophthalmos (HP:0000520), and BOS-like posture. She was born to non-consanguineous parents: a 16-year-old mother and a 22-year-old father, both reportedly healthy. No prenatal care or ultrasound monitoring was conducted. The pregnancy was described as uneventful, with no reported exposure to teratogens, substances of abuse, or infectious diseases.

**Figure 1:**
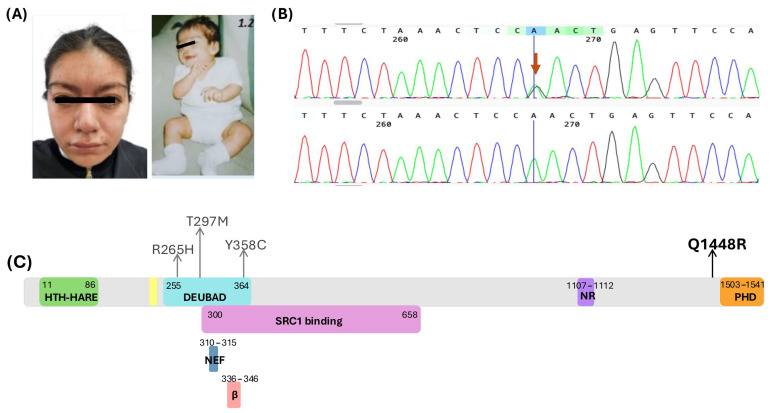
(**A**) Clinical features observed in the patient, highlighting facial characteristics and posture (marked flexion at the elbows and wrists, and ulnar deviation of the hands) consistent with BOS. (**B**) Electropherogram confirming the heterozygous allele of the *ASXL1* variant c.4343A>G (p.Q1448R), identified in the patient (red arrow) and absent in the mother (electropherogram below). (**C**) Schematic representation of the *ASXL1* protein showing the position of the p.Q1448R variant (in bold) and three additional previously reported missense variants (in ClinVar) selected for 3D protein modeling (shown in gray). Major domains, regions and functional motifs are highlighted in different colors (positions based on UniProt ID: Q8IXJ9). α: region involved in interaction with nucleosomal DNA, forming a DNA clamp with BAP1 [17], β: DNA-binding loop with contact to nucleosomal DNA [17], NEF: Asn-Glu-Phe motif that stabilizes the interaction between BAP1 and UBB/ubiquitin [17]. **Abbreviations:** DEUBAD, deubiquitinase adapter domain; NR, nuclear receptor; PHD, plant homeodomain-type zinc finger; HTH-HARE, helix-turn-helix domain found in restriction endonucleases; SRC1, steroid receptor coactivator-1 (also known as nuclear receptor coactivator 1).

She was born at term via vaginal delivery without complications. The birth weight was 2400 g (<1st percentile, Z = −2.25) and length 49 cm (17th percentile, Z = −0.95), head circumference and APGAR scores are unknown, but she cried and breathed spontaneously at birth. Developmental milestones were mildly delayed: social smile at 2 months, head control at 3 months, sitting at 9 months, standing at 11 months, babbling at 12 months, and independent walking at 18 months following physical therapy. In retrospect (after the diagnosis), review of childhood photographs revealed the characteristic Bohring-Opitz syndrome posture, with marked flexion at the elbows and wrists, and ulnar deviation of the hands (Figure 1A).

At 17 years and 2 months, her anthropometric data were: weight 52.5 kg (22nd percentile), height 147.1 cm (<1st percentile, Z = −2.3), and head circumference 52.1 cm (<1st percentile, Z = −2.3). Physical examination revealed an elongated facial shape, with mild facial hirsutism and a vertical forehead crease. She had a broad forehead; thick, arched eyebrows with synophrys tendency; long and prominent eyelashes; mild exophthalmos; lower eyelid distichiasis; upward-slanting palpebral fissures and hypertelorism. The nose was bulbous, with a wide tip and large nares. Other findings included a prominent and wide philtrum, thick lips, marked cupid’s bow (Figure 1A), dental malalignment and retrognathia. Additional findings included a short, broad neck with acanthosis nigricans; a short, wide thorax; Tanner stage IV breast development and a left supernumerary nipple. The abdomen was globose due to adiposity. Genitalia were phenotypically female, Tanner stage IV. The limbs were eutrophic, with mild hypotonia, brachydactyly of the 4th and 5th digits, small hands and feet, absence of interphalangeal creases on the 4th and 5th right fingers, apparent joint hypermobility (Beighton score), and keratosis pilaris. She attended regular elementary education with academic support. She demonstrated partial literacy, basic arithmetic skills, complete self-care, and short-phrase speech with poor articulation. She participated in daily swimming and attended rehabilitation therapy twice weekly.

### 2.2. Ethics Statement

This study protocol was reviewed and approved by the Ethics Committee of the Instituto Nacional de Pediatría, México (293/2025). All procedures performed were in accordance with the ethical standards of the Declaration of Helsinki. Written informed consent was obtained from the patient’s legal guardian for participation in the study, including the collection of a blood sample for whole-exome sequencing and the use of clinical photographs. Additionally, written informed consent has been obtained from the patient’s legal guardian to publish this paper.

### 2.3. Molecular Screening and Variant Interpretation

The patient’s DNA was obtained from peripheral blood leukocytes using a silica-based commercial kit (QIAmp DNA blood Mini Kit, Qiagen, Hilden, Germany), following the manufacturer’s instructions. Genetic testing was performed by whole-exome sequencing (WES). Genomic libraries were prepared using the MGIEasy Exome Capture V5 Probe (MGI Tech Co., Ltd. Shenzhen, China). The libraries were sequenced through massive parallel sequencing (paired-end 2 × 150 bp) using MGI technology. The sequenced data were evaluated for quality control using FastQC program (Version 0.12.0) Simon Andrews. Reads were aligned with BWA (Version 0.7.19) [24] against the human genome version GRCh38. Variant calling was performed with GATK4 (Version 4.6.2.0) [25], assessment of the effects of the variants using SnpEff (Version 5.4.0) [26], and inspection of the selected variants using IGV [27]. The average coverage depth exceeds 99.19% of the targeted regions at a minimum of 20 reads. For *ASXL1*, the sequenced length was 4623 bp (99.94%) with an average depth of 108X. The variant identified in the patient was submitted to LOVD v.3.0. (accession number: 00466058).

### 2.4. In Silico Protein Modeling for Structural Analysis of ASXL1 Variants

To investigate the potential structural consequences of the benign missense variant NM_015338.6:c.4343A>G, p.Gln1448Arg or p.Q1448R identified in our patient, we conducted an in silico structural analysis of the C-term of the ASXL1 protein. Additionally, we partially modeled three other *ASXL1* variants reported as VUS in ClinVar: p.R265H, p.T297M and p.Y358C (Table 1, Figure 1C).

These three variants were selected based on the following criteria: (a) location within an important functional domain [15] as the deubiquitinase adapter domain (DEUBAD); (b) classification as VUS in the ClinVar database [23]; (c) potential disease association indicated in ClinVar; (d) classification as LB or VUS according to ACMG guidelines and Bayesian reinterpretation; (e) present in the GnomAD healthy population database [8,28]; (f) localization within or near a region of the protein for which a crystallographic structure has been previously reported. This analysis aimed to assess possible local conformational changes, disruptions in residue interaction networks, and potential effects on known functional domains of *ASXL1*.

For the p.R265H, p.T297M, and p.Y358C variants, the crystallographic structure of the human polycomb-repressive deubiquitinase (PR-DUB) complex was analyzed. This structure includes BAP1 and ASXL1 bound to the H2AK119Ub nucleosome (PDB ID: 8SVF, chain L; [17]), and served as a reference to evaluate the local structural environment and the potential impact of each amino acid substitution within the ASXL1 region involved in the complex. In addition, for the p.Y358C variant, additional modeling was required that considered a small unresolved segment of ASXL1 in the crystallographic structure. To address this, an extended fragment encompassing residues 347 to 364 was predicted using the AlphaFold2 server [29] and integrated into the 8SVF structure (chain L), enabling a more comprehensive assessment of the substitution’s potential structural consequences.

Due to the absence of crystallographic data for the C-terminal region of ASXL1, the structural impact of the p.Q1448R variant was evaluated using 3D modeling based on the AlphaFold3 server [30]. For this analysis, a C-term segment spanning residues 1441 to 1541 was extracted, as it includes the Q1448 and lies adjacent to a region termed a plant homeodomain (PHD)-type zinc finger. More precisely, this region has been described as an atypical PHD-type zinc finger motif at residues 1503-1540. Accordingly, a zinc ion was incorporated as a second structural entity prior to modeling to accurately reflect the coordination geometry and potential functional implications of this domain with respect to mutation.

All 3D models were visualized and analyzed using PyMOL version 2.5.0 (Schrödinger Inc., New York NY, USA).

## 3. Results

### 3.1. Molecular Screening and Variant Interpretation

The clinical diagnosis of Bohring–Opitz syndrome was suspected by a clinical geneticist based on the patient’s phenotypic features and molecular evaluation. In the in-house bioinformatic analysis of the WES data, we identified a heterozygous variant in *ASXL1* (NM_015338.6:c.4343A>G; p.Q1448R), a gene previously associated with autosomal dominant Bohring–Opitz syndrome. According to ACMG/AMP guidelines and the Bayesian classification framework [1,4], this variant is currently classified as benign (Table 1). Consistently, the p.Q1448R missense variant has been reported in the ClinVar database as benign or likely benign and is present at low allele frequencies in healthy individuals in the gnomAD database (v4.1) [8], supporting a benign interpretation based on population data.

Direct molecular confirmation by Sanger sequencing validated the presence of the c.4343A>G variant in the patient, while the variant was absent in the mother (Figure 1B). The father was not available for genetic testing.

No additional pathogenic, likely pathogenic, or variants of uncertain significance were identified in the whole-exome sequencing analysis, including in genes previously associated with the patient’s clinical phenotype.

### 3.2. In Silico Protein Modeling for Structural Analysis of the ASXL1 p.Q1448R Variant

**Figure 2:**
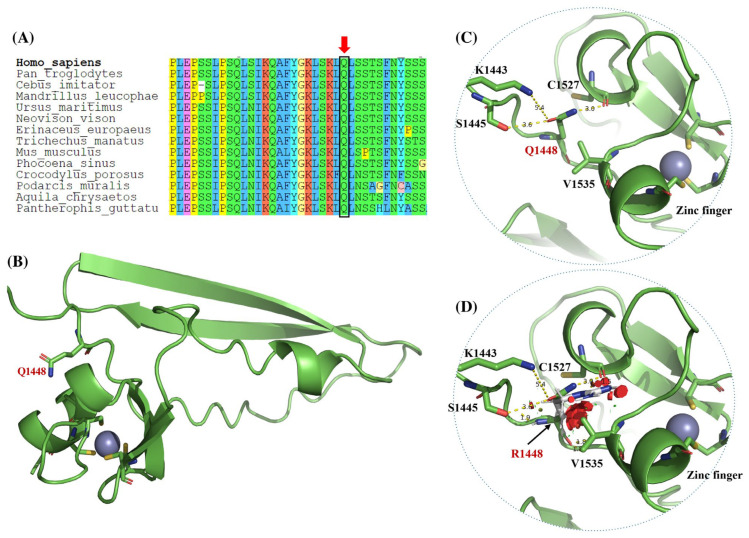
Structural modeling of the p.Q1448R variant in the C-term region of ASXL1 based on AlphaFold3 prediction. (**A**) Multiple sequence alignment showing (red arrow) the location of the mutated amino acid. (**B**) Loops and ribbon representation of the predicted 3D structure of ASXL1 residues 1441–1541, encompassing the C-term region with PHD-type zinc finger domain. It is observed that Q1448 is located near the zinc finger domain. (**C**) Magnified view of the local environment surrounding Q1448, highlighting polar interactions with L1443 (~5.4 Å), S1445 (~3.6 Å), and a hydrogen bond between the NE2 atom of Q1448 and the backbone carbonyl of C1527 (~3.0 Å), which lies within the zinc-binding fold. (**D**) Structural model of the Q1448R variant showing that the substitution of Q with a bulkier, positively charged R side chain disrupts native polar interactions and introduces a potential steric clash with V1535 (~6.0 Å from the zinc-coordinating center). Figure modeled with PyMOL version 2.5.0 (Schrödinger Inc., New York, NY, USA). All structural observations are derived from in silico modeling and represent predicted effects. No experimental functional validation has been performed.

The missense variant p.Q1448R results in the substitution of glutamine by arginine at position 1448 and is located proximal to a predicted PHD-type zinc finger domain spanning residue 1503–1540 (Figure 1C). To evaluate the potential structural consequences of this substitution, we modeled the C-terminal region of *ASXL1* (residues 1441–1541) using AlphaFold3 [30]. The resulting model revealed a canonical PHD zinc finger fold stabilized by a coordinated zinc ion and composed of a double-stranded antiparallel β-sheet structure, consistent with known features of PHD finger domains. Zinc coordination was mediated by conserved cysteine residues, in agreement with previous structural descriptions (Figure 2B).

As shown in Figure 2B,C, residue Q1448 is located adjacent to the PHD-type zinc finger motif within the C-terminal region of ASXL1. In the wild-type (WT) structural model, the side chain of Q1448 participates in a network of stabilizing polar interactions. Thus, Q1448 forms polar contacts with Lys1443 (approximately 5.4 Å) and Ser1445 (approximately 3.6 Å), and its NE2 atom establishes a hydrogen bond with the backbone carbonyl oxygen of Cys1527 (approximately 3.0 Å), a residue within the zinc-binding fold. These interactions are consistent with a probable role for Q1448 in maintaining the local structural organization of the PHD domain.

Upon in silico substitution of glutamine with arginine (Q1448R), the local environment is altered (Figure 2D, missense variant). While spatial proximity to some neighboring residues may still be observed in the static model, the original WT interaction geometry and chemical complementarity are no longer preserved. The longer, positively charged arginine side chain adopts a different orientation, which is predicted to be energetically unfavorable due to the introduction of a steric clash with Val1535, located approximately 6.0 Å from the zinc-coordinating core. Such contacts are predicted to destabilize the native interaction network and, under physiological conditions, likely lead to weakening or loss of the original polar associations observed in the WT structure.

Importantly, because the structural model represents a static snapshot, dynamic rearrangements following the amino acid substitution cannot be directly visualized. Therefore, the inferred loss of native interactions reflects the predicted structural consequences of steric hindrance and altered electrostatics rather than the direct observation of bond rupture. Taken together, comparison of the WT (Figure 2B,C) and Q1448R (Figure 2D) models supports the interpretation that introduction of a bulkier arginine residue may perturb the local structural environment near the PHD domain, providing a plausible structural basis for altered stability or conformational dynamics associated with this missense variant.

### 3.3. Structural Modeling of ASXL1 Variants p.R265H, p.T297M, and p.Y358C

To investigate the potential structural consequences of the *ASXL1* variants p.R265H, p.T297M, and p.Y358C, each classified as a variant of uncertain significance in ClinVar, we performed in silico modeling analyses based on either the crystallographic structure of the human BAP1–ASXL1 complex bound to the H2AK119Ub nucleosome (PDB ID: 8SVF; chain L) [17] or AlphaFold3-predicted models, depending on the availability of resolved structural data.

#### 3.3.1. R265H Variant

**Figure 3:**
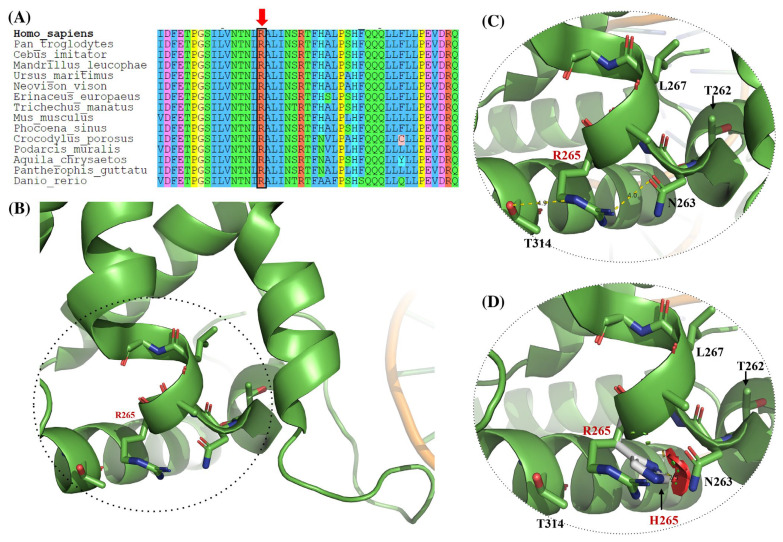
Structural modeling of the p.R265H variant in the ASXL1 crystallographic structure. (**A**) Multiple sequence alignment showing (red arrow) the location of the mutated amino acid. (**B**) Loops and ribbon representation of the local environment surrounding R265 close to DNA (PDB ID: 8SVF, chain L). (**C**) Magnified view of (**B**), where the specific interactions between R265 and its neighboring residues are identified. R265 forms stabilizing noncovalent interactions with the OD1 atom of N263 (~4.0 Å) and the OG1 atom of T314 (~4.9 Å), likely contributing to the local stabilization of the DEUBAD. (**D**) In silico mutation and superposition of the R and H amino acid residues, showing the potential steric clashes generated by the substitution. R265 lies within the DEUBAD (residues 255–364), which is essential for the allosteric activation of BAP1 and the specific deubiquitination of H2AK119Ub. Figure modeled with PyMOL version 2.5.0 (Schrödinger Inc., New York NY, USA). The predicted structural effects are based exclusively on in silico modeling and should be interpreted as hypothesis-generating.

Residue R265 was analyzed using the WT crystallographic structure of ASXL1 (PDB ID: 8SVF). In the WT model (Figure 3B,C), R265 is located near the DNA-binding interface and participates in stabilizing polar interactions. Specifically, the guanidinium group of R265 forms polar contacts with the OD1 atom of Asn263 (approximately 4.0 Å) and the OG1 atom of Thr314 (approximately 4.9 Å). These interactions are indicated by dashed lines and arrows in the zoomed-in panels and are likely to contribute to local structural stability in this region.

In contrast, the missense variant R265H is shown in Figure 3D. Replacement of arginine with histidine alters both side-chain length and charge distribution. In the static structural model, the WT polar interaction network is no longer maintained, and the histidine side chain adopts an alternative orientation. This change introduces potential steric clashes with neighboring residues, highlighted by arrows in the figure, suggesting a localized destabilization of the structural environment adjacent to the DNA-binding interface.

The side-by-side comparison of the WT structure (Figure 3B,C) and the R265H variant (Figure 3D) highlights the potential structural consequences of perturbing this region: substitution of R265 with histidine disrupts stabilizing polar interactions and introduces unfavorable contacts, thereby providing a plausible structural basis for the predicted functional impact of this variant.

#### 3.3.2. T297M Variant

To assess the potential structural consequences of the T297 residue, its local environment was analyzed using the available crystal structure, comparing the WT and missense model.

**Figure 4:**
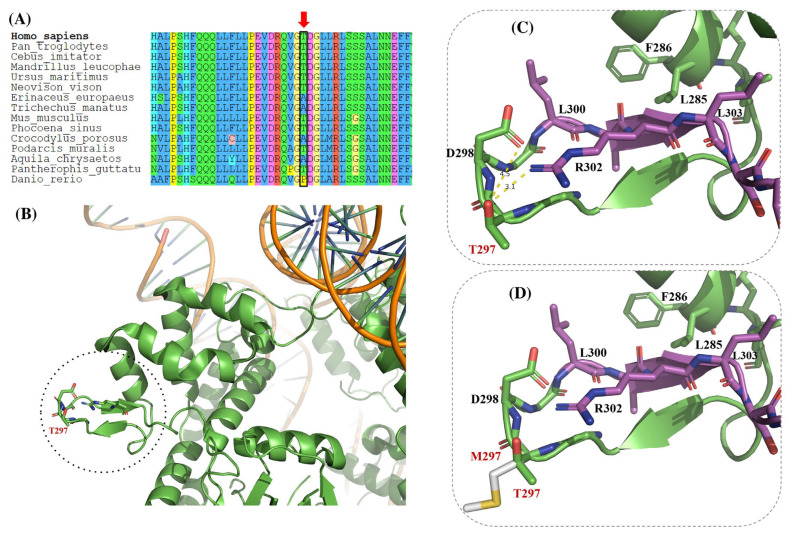
Structural modeling of the p.T297M variant in the crystallographic structure of ASXL1. (**A**) Multiple sequence alignment showing (red arrow) the location of the mutated amino acid. (**B**) Loops and ribbon representation of the ASXL1 region containing T297, located within a solvent-exposed polar loop (PDB ID: 8SVF, chain L). (**C**) Magnified view of (**B**), showing the stabilizing polar interactions formed between the side chain of T297 and the OD1 atom of Asparagine 302 (N302, ~3.1 Å) and the OD2 atom of Aspartic acid 298 (D298, ~4.5 Å). (**D**) In silico modeling of the T297M variant, showing the replacement of Threonine with a bulkier, hydrophobic Methionine side chain, which disrupts the original polar interactions and alters the local microenvironment, the adjacent ASXM/ASXH domain is also indicated in purple. Figure modeled with PyMOL version 2.5.0 (Schrödinger Inc., New York, NY, USA). These observations reflect predicted local structural effects inferred from in silico analysis and lack direct biochemical or functional validation.

Residue T297 is located within a solvent-exposed loop of the DEUBAD, as shown in the WT structural model (Figure 4B,C). In this conformation, threonine at position 297 participates in stabilizing polar interactions with Asp298 (approximately 4.5 Å) and Asn302 (approximately 3.1 Å), indicated by dashed lines in the zoomed-in panels. These interactions are likely to contribute to local loop stability and conformational flexibility.

The T297M missense variant, depicted in Figure 4D, replaces threonine with a bulkier, hydrophobic methionine residue. This substitution abolishes the WT polar interaction network and alters the local microenvironment of the loop, as highlighted by arrows. Although no direct steric clashes are observed in the static model, the change in side-chain chemistry is predicted to perturb local interactions.

Notably, T297 lies immediately adjacent to the conserved ASXM/ASXH domain (residues 300–361), a regulatory element involved in nuclear receptor interactions.

Therefore, the comparison between the WT model (Figure 4B,C) and the T297M variant (Figure 4D) suggests an interesting possibility: the mutation could induce localized structural changes near a critical regulatory motif, providing a plausible structural basis for altered protein–protein interactions.

#### 3.3.3. Y358C Variant

In the case of the residue Y358, its local environment was analyzed using an extended structural model derived from the 8SVF crystal structure, incorporating regions beyond the resolved DEUBAD core and comparing the WT and missense conformation.

**Figure 5:**
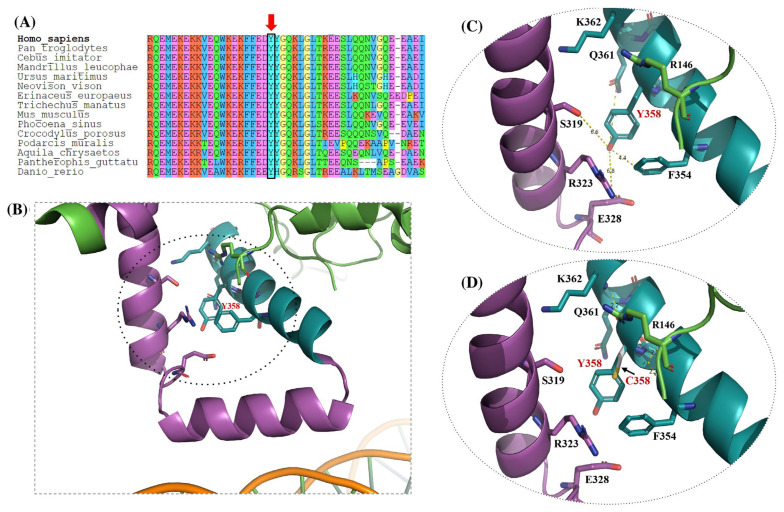
Structural modeling of the p.Y358C variant in the ASXL1 3D structure. (**A**) Multiple sequence alignment showing (red arrow) the location of the mutated amino acid. (**B**) Surface and ribbon representation of a segment of ASXL1, highlighting Y358 within a solvent-accessible segment of the modeled structure based on the PR-DUB complex (PDB ID: 8SVF, chain L). (**C**) Close-up view of panel (**B**), showing the spatial orientation of Y358 relative to its neighboring chain (in purple) and its position relative to the segment involved in direct interaction with nucleosomal DNA. It is observed that Y358 is surrounded by several polar or charged residues: S319, R323, E328, Q361, R146, and F354; most located within ~6 Å, forming a stabilizing hydrophilic microenvironment. (**D**) In silico modeling of the Y358C, illustrating the loss of native polar interactions and the introduction of a smaller, hydrophobic Cysteine side chain. Figure modeled with PyMOL version 2.5.0 (Schrödinger Inc., New York, NY, USA).

In the WT model (Figure 5B,C), tyrosine 358 is positioned within a predominantly polar microenvironment, surrounded by residues S319, R323, E328, Q361, R146, and F354, all located within approximately 6 Å. The hydroxyl group of Y358 contributes to this local polarity and is consistent with a stabilizing role in this region.

In contrast, the Y358C missense variant (Figure 5D) replaces tyrosine with cysteine, resulting in loss of the polar hydroxyl group and introduction of a smaller, more hydrophobic thiol side chain, as indicated by arrows. This substitution could alter the local physicochemical environment without introducing overt steric clashes in the static model.

Although Y358 does not directly contact nucleosomal DNA, it is located adjacent to a solvent-exposed loop implicated in chromatin interaction. Therefore, the side-by-side comparison of the WT (Figure 5B,C) and Y358C variant (Figure 5D) highlights how subtle changes in local polarity could influence ASXL1 function through indirect or allosteric effects.

In Table 2, we summarize the structural localization, native molecular interactions, predicted structural effects, and potential functional implications of the four *ASXL1* missense variants analyzed through in silico modeling.

## 4. Discussion

In this study, we performed a comparative structural analysis of four *ASXL1* missense variants: p.Q1448R, p.R265H, p.T297M, and p.Y358C, to explore how domain-level structural context may inform the interpretation of variants currently classified as benign, likely benign, or of uncertain significance. By integrating in silico protein modeling with evolutionary conservation and domain localization, our findings highlight how subtle perturbations affecting conserved regulatory regions may provide mechanistic insights not captured by population-based or sequence-level predictors alone.

To date, there are more than 2390 reported germline *ASXL1* variants in ClinVar [23]. Notably, ~53% are VUS or show conflicting interpretations, ~41% are benign or likely benign, and only ~6% are pathogenic or likely pathogenic. This distribution illustrates the interpretative challenge, particularly since *ASXL1* is also frequently affected by somatic mutations due to clonal hematopoiesis of indeterminate potential, especially in aging individuals [10,12,31]. The presence of such somatic variants in reference databases such as gnomAD can lead to misinterpretation during germline variant analysis, a concern that has been previously documented in BOS studies [13,14]. This dual role of *ASXL1* in developmental disorders and age-related clonal expansion highlights a structural limitation of population databases for variant interpretation, particularly for genes involved in chromatin regulation and hematopoiesis. Because ACMG/AMP classification frameworks rely in part on population frequency evidence, the inclusion of somatic variants in reference datasets may, in certain contexts, contribute to the classification of potentially relevant germline variants as benign or likely benign.

Importantly, a high proportion of missense variants classified as VUS is not unique to *ASXL1* and has been observed in other autosomal dominant disorders, including those traditionally associated with truncating pathogenic mechanisms. Recent large-scale functional and structural studies have shown that genes with complex domain architectures and extensive protein–protein interactions often accumulate substantial numbers of missense variants with uncertain interpretation. For example, comprehensive functional landscapes generated for clinically relevant genes demonstrate that a large fraction of missense variants initially classified as VUS exhibit measurable functional effects only detectable through systematic experimental or structural approaches [32,33]. These findings indicate that the high prevalence of VUS in *ASXL1* reflects broader challenges in variant interpretation rather than an exceptional feature of this gene alone.

In this context, the interpretation of apparent homozygosity in population databases warrants caution for genes frequently involved in clonal hematopoiesis. Studies in myeloid malignancies have shown that variant allele frequencies (VAFs) can span a wide range, from low-level subclonal events to near-homozygous states resulting from clonal expansion, loss of the wild-type allele, or acquisition of multiple mutations within the same gene. In *ASXL1*, reported VAFs in acute myeloid leukemia range from ~1% to >50%, consistent with heterozygous mutations, while other clonal hematopoiesis–associated genes such as *DNMT3A*, *TET2*, *JAK2*, and *TP53* frequently exhibit VAFs exceeding 90%, reflecting near-homozygous states [34]. Although less common, similar mechanisms have been reported for *ASXL1*, indicating that apparent homozygosity in sequencing datasets does not unequivocally indicate germline inheritance or benignity, particularly when derived from blood-based samples.

The p.Q1448R variant identified in our patient is classified as likely benign under ACMG/AMP guidelines and Bayesian classification framework [1,4] and is present in gnomAD (rs772452614) [8], with both heterozygous and one homozygous carrier. Despite this classification, the variant was identified in a heterozygous state and allegedly *de novo*, in a female patient with clinical features compatible with BOS, motivating additional analyses beyond conventional criteria. In silico structural modeling showed that this variant, located adjacent to a PHD-type zinc finger domain, abolishes stabilizing polar contacts and introduces steric hindrance near the zinc-coordinating site. The PHD finger (residues 1503–1540) is an evolutionarily conserved structural module involved in histone recognition and chromatin remodeling [19]. Our model suggests that the p.Q1448R variant may impair the local structure and disrupt histone mark recognition, which could inform future reclassification.

Moreover, three additional variants: p.R265H, p.T297M, and p.Y358C, were studied using available crystallographic and AlphaFold-based structures. All are located within or near the DEUBAD (residues 255–364), which interacts with the ULD domain of BAP1 to form a ubiquitin binding cleft essential for the function of the PR-DUB complex [15,17]. The p.R265H variant disrupts local polar contacts and lies close to key catalytic residues (e.g., L267, T262), whose substitution has been shown to severely impair deubiquitination activity without affecting nucleosome binding [17].

Particularly, the p.T297M variant, located in a solvent-exposed loop within the DEUBAD, likely disrupts flexibility and loop structure. It is located adjacent to the nuclear receptor (NR) interaction motif (residues 299–303), which mediates interactions with nuclear coactivators such as NCOA1 [35]. By introducing a hydrophobic residue, this variant may interfere with transcriptional coactivation, suggesting dual functional impacts on both chromatin remodeling and nuclear receptor signaling. In the case of p.Y358C, although this residue does not directly contact nucleosomal DNA, it is located near a loop that mediates PR-DUB interaction with chromatin [17]. The replacement of tyrosine with cysteine eliminates key polar interactions and may induce local misfolding or destabilization, potentially interfering allosterically with nucleosome binding. 

Furthermore, all modeled variants, except for the p.T297M, are located within ultraconserved regions across vertebrate species, suggesting they lie in sites of strong evolutionary constraint and low tolerance for change. This is particularly relevant for p.Q1448R, which lies near the PHD zinc finger, a domain widely conserved among the ASXL family proteins and chromatin-associated proteins [18,36]. PHD fingers are known to interpret specific histone marks and regulate transcription by mediating interactions with the nucleosome.

Recent large-scale functional studies support the relevance of this structural context. Systematic functional landscapes of missense variation have demonstrated that variants affecting conserved structural microenvironments, rather than catalytic residues alone, can exert significant functional effects, often missed by traditional in silico predictors [33]. These findings are particularly relevant for multifunctional chromatin regulators such as ASXL1, where subtle perturbations in domain architecture or interaction surfaces may lead to downstream transcriptional dysregulation.

Importantly, the structural analyses presented here are not intended to establish pathogenicity or functional impairment per se, but rather to provide domain-level, hypothesis-generating insights that complement existing population-based and computational evidence. In the absence of experimental validation, these models should be interpreted as indicative of potential structural sensitivity rather than definitive functional disruption.

Functionally, the PR-DUB complex is composed of a catalytic core (BAP1 + ASXL1/2/3 + MBD5/6) and several accessory proteins such as FOXK1, KDM1B, and NCOA1 [16,19]. The DEUBAD of ASXL1 stabilizes the ULD domain of BAP1 and aligns the active site with its H2AK119Ub substrate [17]. Structural alterations in these domains such as those predicted for p.R265H and p.T297M may interfere with catalytic efficiency, coactivator recruitment, or chromatin engagement, and ultimately compromise transcriptional regulation and hematopoietic differentiation [15,20]. Therefore, our structural models suggest that missense variants currently classified as benign/likely benign or VUS, such as p.Q1448R, could have pathogenic effects if they disrupt the structural and functional microenvironments of critical domains. However, *in silico* approaches should be interpreted as hypothesis-generating, as computational predictors alone have limited sensitivity and specificity for variant classification in complex proteins [33].

As summarized in Table 2, the analyzed *ASXL1* missense variants affect conserved domains and residue interaction networks that are critical for chromatin regulation, including the DEUBAD and PHD domains. Although all four variants were previously classified as benign/likely benign or VUS, the structural alterations observed, particularly those involving residues adjacent to key regulatory interfaces, support potential mechanistic roles in modulating ASXL1 protein function. These observations reinforce the importance of complementing population-based and computational evidence with structural context and, ultimately, experimental functional data when interpreting *ASXL1* missense variants.

In this context, multiplexed assays of variant effect (MAVEs) represent an emerging gold standard for functional interpretation. Studies in clinically relevant genes have shown that MAVEs can reclassify a substantial fraction of VUS by providing quantitative functional evidence across thousands of variants [32]. Curated resources such as ClinMAVE and MaveMD further illustrate how functional datasets can be translated into clinical variant interpretation frameworks and integrated with ACMG/AMP criteria [37,38]. Although *ASXL1*-specific MAVE data are not yet available, these approaches provide a conceptual and methodological roadmap for resolving uncertainty in genes with complex structure–function relationships.

### Study Limitations and Future Directions

This study provides a detailed structural and mechanistic framework to interpret *ASXL1* missense variants in the context of Bohring–Opitz syndrome. While based on a single clinical case, the depth of molecular modeling and comparative domain-level analysis offers insights that extend beyond individual variant description. As with most studies relying on in silico approaches, functional hypotheses generated here will ultimately benefit from experimental validation. However, the structural analyses presented illustrate how three-dimensional context can uncover potential pathogenic mechanisms that are not captured by current population-based or computational classifiers. The coexistence of germline and somatic variation in *ASXL1* remains an important consideration for variant interpretation and underscores the need for gene-specific frameworks that integrate clinical, structural, and functional data.

Looking forward, the systematic generation of *ASXL1*-specific functional datasets, including MAVE, represents a natural extension of the approach presented here. Integration of such functional evidence with longitudinal clinical data and international data-sharing efforts will be essential to refine variant classification and to translate mechanistic insights into improved clinical decision-making.

## 5. Conclusions

This study highlights the value of integrating in silico structural modeling as a complementary approach for interpreting *ASXL1* missense variants in the context of BOS. Through comparative analyses of four variants: p.Q1448R, p.R265H, p.T297M, and p.Y358C, we identified predicted local structural perturbations affecting conserved and functionally relevant domains, including the DEUBAD region and the PHD-type zinc finger.

Our findings underscore the limitations of relying exclusively on population frequency data and standard computational predictors for variant interpretation in genes such as *ASXL1*, which are involved in both germline developmental disorders and somatic clonal hematopoiesis. Incorporating domain-level structural context may provide additional mechanistic insight and help prioritize variants for further evaluation.

Rather than supporting definitive reclassification, the structural analyses presented here are intended as hypothesis-generating evidence. As functional datasets and high-throughput assays become increasingly available, integrative approaches combining clinical information, structural modeling, and functional validation will be essential to refine variant interpretation and improve molecular diagnosis in *ASXL1*-related disorders.

## Figures and Tables

**Table 1:** Summary of *ASXL1* (NM_015338.6) benign, LB and VUS variants analyzed by 3D protein modeling.

Variant (dbSNP)	Location	ClinVar Accession (Classified as) [23]	ClinVar Associated Condition	ACMG/AMP Classification †	GnomAD Variant ID, Allele Frequencies [8]
c.4343A>G, p.Q1448R (rs772452614)	Near PHD (Zinc finger)	VCV001302809.15 (B, LB)	BOS, *ASXL1*-related disorder, Inborn genetic disease	Benign: −21 points (BS1, BS2, BP1, BP4, BP6)	20-32437055-A-G, Total: 0.00003098 AMX: 0.0007500
c.794G>A p.R265H (rs144349534)	DEUBAD	VCV001393725.7 (VUS)	Not Reported	LB: −5 puntos (BS2, BP1, PP3)	20-32431396-G-A, Total: 0.00003779 AMX: 0.00001667
c.890C>T p.T297M (rs373599045)	DEUBAD	VCV001375283.7 (VUS)	Not Reported	LB: −1 points (1P-2B)	20-32431590-C-T, Total: 0.00001487 AMX: 0.000
c.1073A>G p.Y358C (rs1415471931)	DEUBAD	VCV002439274 (VUS)	BOS	VUS: 0 puntos (PM2, BP1)	20-32432973-A-G, Total: 0.000003718 AMX: 0.00003333

**Abbreviations:** AMX, admixed American; BOS, Bohring–Opitz syndrome; ID, identification; LB, likely benign; PHD, plant homeodomain (zinc finger); VUS, variant of uncertain significance. † According to ACMG/AMP guidelines and Bayesian classification.

**Table 2:** Summary of predicted structural context, native molecular interactions, and inferred local structural effects of four *ASXL1* missense variants based on in silico modeling.

Variant	Structural Location	Native Interactions (WT Model)	Predicted Local Structural Effect (In Silico)	Inferred Functional Implication (Hypothesis-Generating)
p.Q1448R	Adjacent to the PHD-type zinc finger domain	Polar contacts with Lys1443 (~5.4 Å) and Ser1445 (~3.6 Å); hydrogen bond with the backbone carbonyl of Cys1527 (~3.0 Å)	Disruption of native polar interaction geometry; introduction of a steric clash with Val1535 (~6 Å from the zinc-coordinating core)	Local destabilization of the PHD zinc finger environment; potential impairment of chromatin recognition or domain stability
p.R265H	DEUBAD, near the DNA-interacting interface	Polar contacts with Asn263 (~4.0 Å) and Thr314 (~4.9 Å)	Loss of stabilizing polar interactions; altered side-chain orientation with potential unfavorable contacts	Possible destabilization of the DEUBAD and altered PR-DUB catalytic activity or chromatin engagement
p.T297M	Solvent-exposed loop of the DEUBAD, adjacent to the LXXLL motif	Polar interactions with Asp298 (~4.5 Å) and Asn302 (~3.1 Å)	Abolishment of polar contacts; introduction of a bulkier hydrophobic side chain in a polar environment	Potential perturbation of loop flexibility and regulatory interactions, possibly affecting nuclear receptor–mediated transcriptional regulation
p.Y358C	Polar microenvironment near a solvent-exposed loop implicated in chromatin interaction	Surrounded by polar residues (Ser319, Arg323, Glu328, Gln361, Arg146, Phe354; within ~6 Å)	Loss of the tyrosine hydroxyl group; reduced local polarity without overt steric clashes	Subtle alteration of local physicochemical properties; possible indirect or allosteric effects on DNA- or nucleosome-associated interactions

## Data Availability

The original contributions presented in this study are included in the article. The variant in *ASXL1* that was identified in the patient was submitted and is available at the Leiden Open Variation Database, LOVD3 (https://databases.lovd.nl/shared/genes/ASXL1), accessed on 05 January 2026. Further information can be available after inquiries to the corresponding author.

## Abbreviations

The following abbreviations are used in this manuscript:

ACMG	American College of Medical Genetics and Genomics
AML	Acute myeloid leukemia
AMP	Association for Molecular Pathology
AMX	Admixed American
BOS	Bohring–Opitz Syndrome
CHIP	Clonal hematopoiesis of indeterminate potential
CMML	Chronic myelomonocytic leukemia
DEUBAD	Deubiquitinase adapter domain
HTH-HARE	Helix-turn-helix domain found in restriction endonucleases
ID	Identification
LB	Likely benign
MAVE	Multiplexed Assays of Variant Effect
MDS	Myelodysplastic Syndromes
MPN	Myeloproliferative neoplasms
NGS	Next-generation sequencing
NR	Nuclear receptor
PHD	Plant homeodomain-type zinc finger
PR-DUB	Polycomb repressive deubiquitinase
SRC1	Steroid Receptor Coactivator-1
VAFs	Variant allele frequencies
VUS	Variants of uncertain significance
WES	Whole-exome sequencing
